# Editorial: AI and resilience

**DOI:** 10.3389/frai.2026.1887642

**Published:** 2026-06-23

**Authors:** Ekkehard Ernst, Nicholas A. Friedenberg, Eleni Nisioti, Kaushik Das, Emily Gates, Ilvanna Salas, Mona Sloane

**Affiliations:** 1 International Labour Organization, Geneva, Switzerland; 2 Corteva Agriscience, Indianapolis, IN, United States; 3 Robotics, Evolution and Art Lab, IT-Universitetet i Kobenhavn, Copenhagen, Denmark; 4 Peregrine Data, San Franscico, CA, United States; 5 School of Education and Human Development, Boston College, Chestnut Hill, MA, United States; 6 Universidad de Los Andes, Merida, Venezuela; 7 School of Data Science, University of Virginia, Charlottesville, VA, United States

**Keywords:** adaptability, algorithmic management, artificial intelligence, climate change, complexity, global governance, macroeconomic (in-)stability, resilience

Coming from a wide variety of disciplines, our editorial team gave itself the task to explore how the concept of “resilience,” ambiguous and contested as it is, might help improve our understanding of the role of artificial intelligence (AI) in addressing some of the most pressing global issues. Rather than viewing AI narrowly within a specific field and domain, our multi-disciplinary editorial board aimed at looking at cross-cutting issues that warrant a wider perspective. Climate disruption and pandemic-driven labor upheavals, financial fragility and contested governance, all demand tools to enhance the capacity of a system to absorb shocks while preserving its core functions, which is what we took as our working definition of resilience. Thanks to its capacity to rapidly process vast volumes of—often unstructured—information and to issue predictions to guide behavior, AI is frequently presented as a candidate for that role. Yet, as the seven contributions to this Research Topic make clear, the relationship between AI and resilience is anything but straightforward. AI can extend a system's adaptive capacities, but it can also introduce new sources of brittleness, exposing operators, workers and citizens to opaque, unaccountable forms of disruption and new forms of harm. It can forecast climate stress, simulate sovereign debt trajectories, or coordinate fragmented policy responses, but can also generate new vulnerabilities, opacity, and asymmetries of power. The seven contributions to this Research Topic probe both sides of this dynamic across domains as varied as climate adaptation, macroeconomic policy, platform labor, cybersecurity, global governance, and the topology of cooperation itself.

## AI as an enabler of resilience

Three contributions examine AI as an instrument deployed to design for resilience. Ayadi et al. conduct a systematic review of 385 peer-reviewed studies on AI for climate resilience, mapping a landscape skewed toward adaptation over mitigation and dominated by classical machine learning. They find agriculture and urban infrastructure to be the best-served sectors, while Africa and South America remain underrepresented, raising urgent questions about equitable deployment. Khundadze and Semmler complement this picture with a tightly focused empirical exercise: applying reinforcement learning to Eurozone sovereign debt management. Their simulations show that AI-driven optimization can support cooperative monetary and fiscal coordination, the absence of which fuelled both the 2009–2012 Eurozone crisis and the disruptions of the COVID-19 period. Castro-Gonzalez et al. zoom out further, modeling cooperation as a network-dependent property of agents playing a stag-hunt game with memory; their findings show that the topology of interactions, not individual rationality alone, determines whether cooperative behavior survives a shock.

## AI as a source of shocks

A second cluster of articles confronts AI itself as a source of shock and brittleness. Williams and Rani synthesize a decade of ILO research on digital labor platforms to argue that algorithmic management functions are a shock to worker wellbeing, against which resilience must be actively built through informal and formal acts of resistance, often mediated by the very social media platforms that workers depend on for income. Donoghue extends this analysis philosophically, identifying a “foresight endangerment problem” in algorithmic management: as algorithms become more resilient and useful, their outputs grow more opaque, eroding the worker's ability to anticipate the consequences of her own choices. He calls this the resilience-predictability paradox, a striking formulation that ought to guide design choices well-beyond the gig economy. Radanliev et al. extend the brittleness argument to the technology itself, presenting a five-stage life-cycle framework for the cybersecurity governance of generative AI and warning that current oversight mechanisms are inadequate to contain risks ranging from deepfake-driven misinformation to adversarial manipulation of model behavior.

## Bridging frames: governance and complexity

The seventh contribution, by Ilcic et al., integrates these strands within a complexity-science framing of global governance. Treating AI as an embedded epistemic agent rather than a neutral tool, they argue that adaptive, modular and inclusive governance arrangements, informed by complexity insights, are the only credible response to AI's dual capacity for transformation and disruption. Their account makes explicit what is implicit across the issue: resilience cannot be designed from a single vantage point, and any attempt to engineer it through AI must reckon with the interdependence of technical, economic, social, and ethical scales.

**Figure 1:**
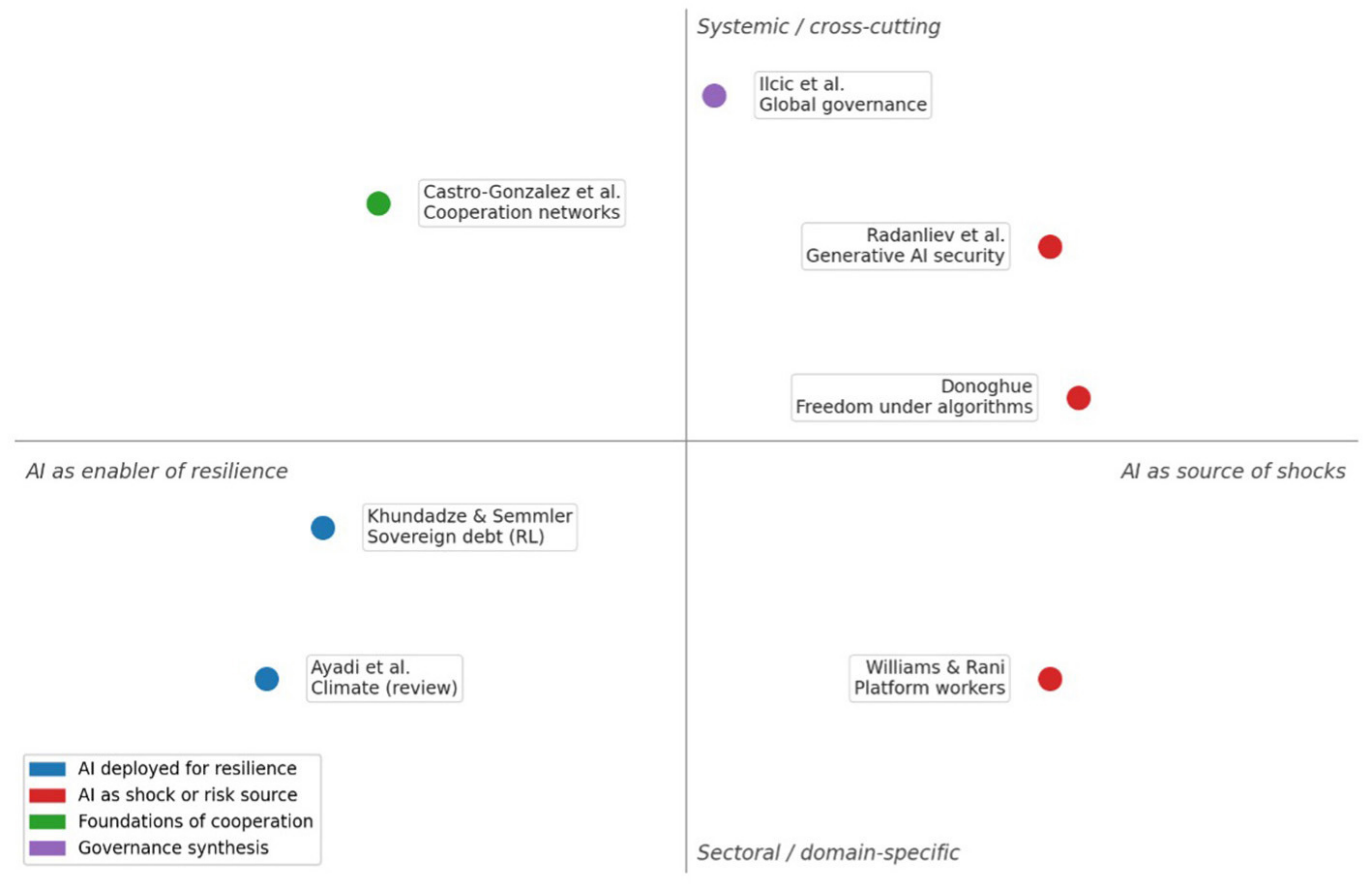
Positioning the seven contributions in the AI-resilience space.

Read together, these contributions speak directly to the objectives that motivated this Research Topic (see Figure 1). Three specific insights emerge. First, AI is best understood as a double-edged instrument: it can enhance forecasting, coordination and stress-testing while simultaneously introducing new opacity, brittleness and concentrations of control. The papers by Donoghue and Radanliev et al. make this duality concrete, while Ilcic et al. provide the conceptual scaffolding to think about it. Second, the design of AI matters as much as its deployment. The choice between an explainable model and a black-box optimiser is not a technical detail but a determinant of who can foresee, contest and adapt to algorithmic decisions. Third, resilience is irreducibly relational. Whether through worker solidarity (Williams and Rani), policy cooperation (Khundadze and Semmler), or network topology (Castro-Gonzalez et al.), the structure of connections among agents, rather than any individual capability, determines whether shocks are absorbed or amplified. By extension, AI may influence societal resilience through its effect on network topology and its mediation of “saving traits” that promote cooperation.

## Looking ahead

Read together, the seven articles map the contours of an emerging research programme rather than offering a settled doctrine. They include theoretical, empirical and aspirational contributions: empirical case studies (Williams and Rani; Donoghue), theoretical and computational modeling (Khundadze and Semmler; Castro-Gonzalez et al.), systematic syntheses (Ayadi et al.; Radanliev et al.), and normative-conceptual work (Ilcic et al.). Looking ahead, further research should close remaining gaps. The use of AI in regulatory stress testing receives detailed attention chiefly in the macroeconomic domain, while feedback loops between AI-mediated information and collective behavior, identified at the outset as a frontier topic, are explored mainly through the lens of platform labor. We believe these feedback loops should be analyzed more broadly as AI permeates all forms of social life. As editors and academic researchers, we hope having demonstrated the importance of a multi-disciplinary approach that stimulates future contributions. We do consider it essential to integrate a variety of perspectives into actionable policies and regulation, examine the regional and distributional implications of AI-mediated resilience, and engage seriously with the question of whose resilience AI is ultimately serving. We also think that future work would benefit from more participatory case studies across a larger range of countries, and from sustained attention to the political economy of AI-enabled resilience. The articles assembled here suggest that getting these answers right will require sustained cross-disciplinary dialogue and engagement between engineers, social scientists, regulators and the communities most exposed to the shocks that resilience is meant to absorb.

